# The road to resistance

**DOI:** 10.7554/eLife.52092

**Published:** 2019-10-25

**Authors:** Devon M Fitzgerald

**Affiliations:** 1 Department of Molecular and Human GeneticsBaylor College of MedicineHoustonUnited States; 2 Department of Biochemistry and Molecular BiologyBaylor College of MedicineHoustonUnited States; 3 Department of Molecular Virology and MicrobiologyBaylor College of MedicineHoustonUnited States; 4 Dan L Duncan Comprehensive Cancer CenterBaylor College of MedicineHoustonUnited States

**Keywords:** *Acinetobacter baumanni*i, biofilm, population genetics, antimicrobial resistance, bacterial evolution, Other

## Abstract

The way that bacteria grow – either floating in liquid or attached to a surface – affects their ability to evolve antimicrobial resistance and our ability to treat infections.

**Related research article** Santos-Lopez A, Marshall CW, Scribner MR, Snyder DJ, Cooper VS. 2019. Evolutionary pathways to antibiotic resistance are dependent upon environmental structure and bacterial lifestyle. *eLife* **8**:e47612. doi: 10.7554/eLife.47612

Think about the last time you or a loved one had a bacterial infection and needed antibiotics. What if the antibiotics hadn’t worked? Unfortunately, this is becoming increasingly common around the world, causing at least 700,000 deaths per year (O’Neill, 2016). Resistance to antimicrobial treatments, also known as AMR, evolves rapidly, often over the course of a single infection. It occurs either by bacteria exchanging with one another the genes that confer resistance, or by an individual bacterium becoming resistant through mutations in its own genes. Resistant bacteria are more likely to survive antimicrobial treatments and go on to spread the infection to other people.

During infections, bacteria tend to grow on surfaces in communities called biofilms (Koo et al., 2017). Biofilms promote communication and cooperation, and physically shield bacteria from antimicrobials and the host immune system. These properties make biofilm infections hard to treat, even in the absence of AMR. It has been suggested that bacteria growing in biofilms may evolve AMR more rapidly, making treatment outcomes harder to predict. However, few studies have explored how different modes of growth influence the ability of bacteria to evolve (Steenackers et al., 2016).

Now, in eLife, Vaughn Cooper and colleagues at the University of Pittsburg — including Alfonso Santos-Lopez and Christopher Marshall as joint first authors — report how 'bacterial lifestyle' affects the evolution of resistance to an antibiotic called ciprofloxacin (CIP) in the pathogen *Acinetobacter baumannii* (Santos-Lopez et al., 2019). To do this, they compared bacteria that had been floating in liquid culture as they grew to bacteria that had been growing as biofilms. The bacteria growing in liquid culture were experimentally evolved by transferring small amounts of culture to a new container with fresh growth medium every day (Figure 1A). Biofilms were evolved by growing *A. baumannii* on plastic beads submerged in liquid culture, and transferring these biofilm-coated beads to a new container with fresh medium and a clean bead each day (Figure 1B). At the start of the experiment, cells were exposed to a very low dose of CIP, and the CIP dose was then doubled every 72 hours as resistance evolved over a 12 day period.

**Figure 1.**
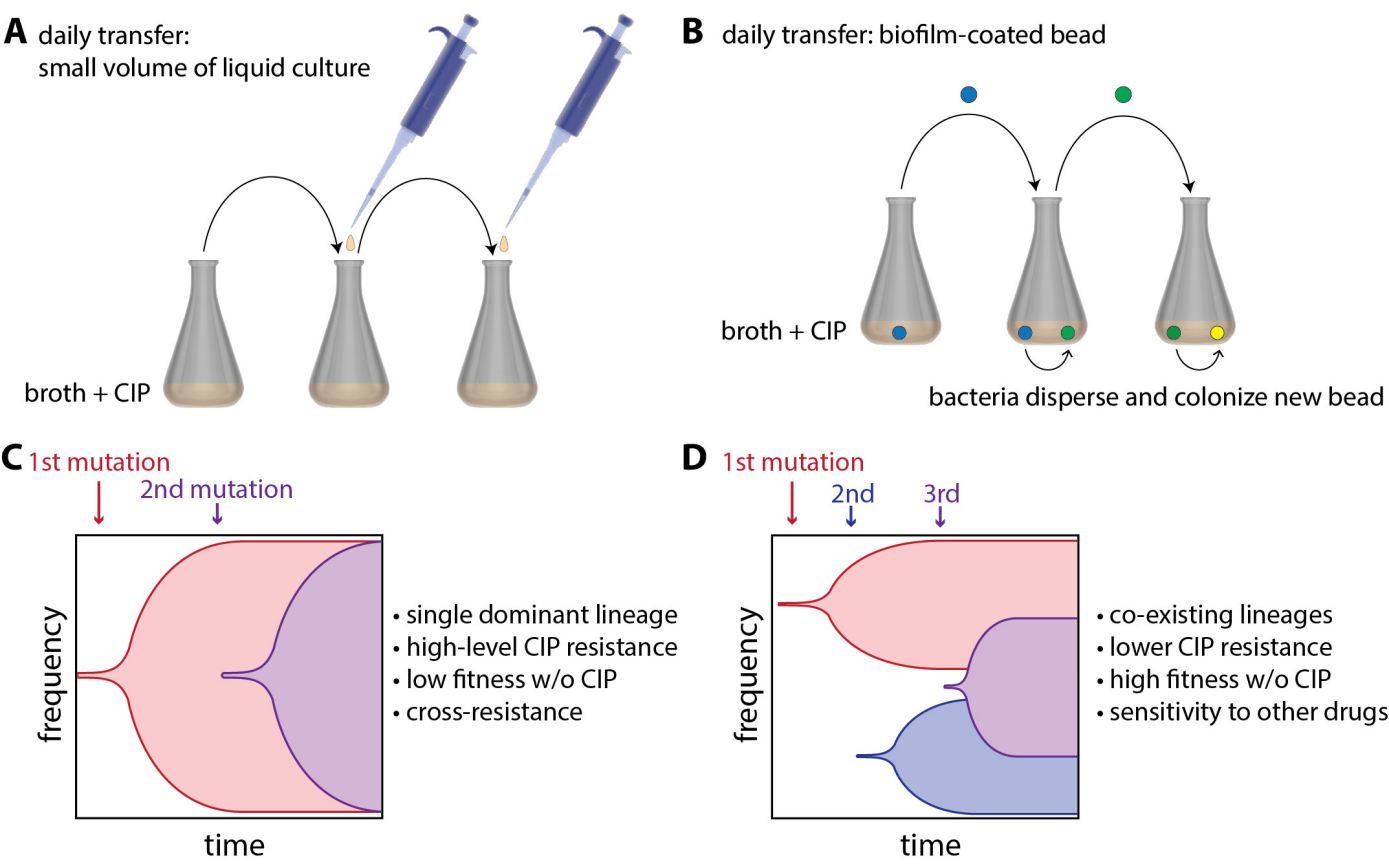
Experimental evolution of resistance to ciprofloxacin (CIP) in liquid-grown and biofilm-grown bacteria. To understand how bacterial lifestyle influences the evolution antimicrobial resistance, strains of the bacterium *A.baumannii* were experimentally evolved either in liquid culture (**A**) or as biolfims on plastic beads (**B**). Santos-Lopez et al. found that liquid-grown and biofilm-grown strains of *A.baumannii* evolved resistance to the antimicrobial CIP in different ways: liquid-grown bacteria evolved a single resistant lineage that dominated the population (**C**), whereas biofilm-grown bacteria evolved multiple resistant lineages that co-exist within the population (**D**). Other important characteristics of the two evolved strains (such as resistance to CIP) are discussed in the main text.

Samples of bacteria were collected from each culture at various times during the experiment and also at the end of the experiment. Whole genome sequencing of these samples revealed that liquid-grown and biofilm-grown *A. baumannii* take different, but repeatable, paths during experimental evolution. For the bacteria grown in liquid culture, Santos-Lopez et al. found that the most resistant CIP lineage outcompetes the other bacterial lineages, allowing its offspring to rapidly take over the population (Figure 1C). However, for the bacteria grown in biofilms, the researchers found that there was less competition between lineages – presumably because they are not as free to move as the liquid-grown bacteria – and that multiple co-existing lineages were able to emerge (Figure 1D).

Further experiments showed that liquid-grown and biofilm-grown bacteria had evolved unique strategies to resist CIP, resulting in different degrees of CIP resistance. Liquid-grown bacteria evolved low-level CIP resistance via mutations that increased the number of pump proteins that transport CIP out of cells. These resistant cells took over rapidly and then gained additional mutations that prevented CIP from binding to its target proteins. Bacteria with both types of mutations could resist doses of CIP that were far higher than those used during the evolution experiment. Biofilm-grown bacteria also evolved low-level CIP resistance through increased pump protein production but used different pumps than their liquid-evolved counterparts. However, biofilm-grown bacteria failed to evolve high-level resistance through additional mutations. The selective pressures imposed by the different lifestyles seem to interact with the pressure of CIP exposure and limit the evolutionary pathways, and thus outcomes, available to each lifestyle.

Santos-Lopez et al. found that in the absence of CIP, liquid-evolved CIP resistant bacteria were less fit than the ancestor strain and could be easily outcompeted. Notably, similar fitness trade-offs have been reported before and reduced fitness is thought to slow the spread of AMR bacteria. Disturbingly, biofilm-evolved CIP-resistant strains were at least as fit as their ancestor when grown without CIP. Whilst the liquid-evolved strain frequently became resistant to additional antimicrobials, biofilm-grown bacteria usually evolved increased sensitivity to at least one other antimicrobial treatment. This means that although biofilm-evolved strains may be fitter and more likely to spread to new patients, they also may be easier to kill with other antimicrobials.

In this work, Santos-Lopez et al. have focused on how biofilms affect competition and selection pressures during evolution. However, biofilms may alter evolution in other ways and bacterial lifestyle is only one of many factors that shape the evolution of AMR 'in the wild.' For example, many species of bacteria experience increased gene sharing and mutation rates when forming a biofilm, both of which could also drive the evolution of AMR (Steenackers et al., 2016). Additionally, AMR evolution may be driven by increases in mutation rate caused by antimicrobials (Cirz et al., 2005; Kohanski et al., 2010; Gutierrez et al., 2013; Pribis et al., 2019) and other stressors, such as starvation, encountered by bacteria during infection (Fitzgerald and Rosenberg, 2019).

These findings have important implications for treatment of *A. baumannii,* which is already resistant to multiple drugs and has been deemed a critical threat by both the World Health Organization and the Centers for Disease Control and Prevention in the United States. A better understanding of the variables shaping AMR evolution will improve predictions of evolutionary outcomes, antimicrobial stewardship efforts, and clinical outcomes for individual patients.
